# A Review on the Marek’s Disease Outbreak and Its Virulence-Related *meq* Genovariation in Asia between 2011 and 2021

**DOI:** 10.3390/ani12050540

**Published:** 2022-02-22

**Authors:** Baolin Song, Jehan Zeb, Sabir Hussain, Muhammad Umair Aziz, Elena Circella, Gaia Casalino, Antonio Camarda, Guan Yang, Nicolas Buchon, Olivier Sparagano

**Affiliations:** 1 Department of Infectious Diseases and Public Health, Jockey Club College of Veterinary Medicine and Life Sciences, City University of Hong Kong, Kowloon, Hong Kong 999077, China; baolin.song@my.cityu.edu.hk (B.S.); jehanzeb2@cityu.edu.hk (J.Z.); sahussain8-c@my.cityu.edu.hk (S.H.); muhamaziz3-c@my.cityu.edu.hk (M.U.A.); gyang25@cityu.edu.hk (G.Y.); 2 Department of Veterinary Medicine, University of Bari, S.P. Casamassima km. 3, 70010 Valenzano, Italy; elena.circella@uniba.it (E.C.); gaia.casalino@uniba.it (G.C.); antonio.camarda@uniba.it (A.C.); 3 Department of Entomology, Cornell Institute of Host-Microbe Interactions and Disease, Cornell University, Ithaca, NY 14853, USA; nicolas.buchon@cornell.edu

**Keywords:** Marek’s disease, virus, Asia, pathology, *meq* sequence

## Abstract

**Simple Summary:**

Marek’s disease is continuously causing an economic loss in Asia, despite the wide use of vaccines in the last decade. This review aims at summarizing the outbreak, the virulence-related *meq* gene variation, and the pathological information of Marek’s disease in the last decade in Asia. We found that a total of 132 viral strains emerged in 12 countries with different meq sequences. Among the evidence we have collected, 12 strains found in China were vaccine-resistant, reaching a mortality rate of 30% and above. This evidence requires the related region in China to consider the renewal of its vaccination type; however, more studies regarding the vaccination efficiency in other Asian countries are recommended, as the current information is not enough. The visceral tumor is the most common pathological type (13 in 16 studies) in Asia, while it is possible that a neural type may exist. We suggest that farmers monitor the behavioral changes of chickens to identify this harmful disease at the early stage. The phylogenetic analysis shows interconnection between Middle Eastern, South Asian, and East Asian countries that are geologically connected—poultry trading managers should consider the potential of viral transmitting.

**Abstract:**

Marek’s disease is an infectious disease in poultry that usually appears in neural and visceral tumors. This disease is caused by *Gallid alphaherpesvirus 2* infection in lymphocytes, and its meq gene is commonly used in virulent studies for coding the key protein functional in oncogenic transformation of the lymphocytes. Although vaccines have been introduced in many countries to control its spread and are proven to be efficient, recent records show a decline of such efficiency due to viral evolution. In this study, we reviewed the outbreak of Marek’s disease in Asia for the last 10 years, together with associated *meq* sequences, finding a total of 36 studies recording outbreaks with 132 viral strains in 12 countries. The visceral type is the most common (13 in 16 studies) form of Marek’s disease, but additional unobserved neural changes may exist. MD induces liver lymphoma most frequently (11 in 14 studies), and tumors were also found in spleen, kidney, heart, gizzard, skin, intestine, lung, and sciatic nerve. Twelve viral strains distributed in China have been reported to escape the CVI988 vaccine, reaching a mortality rate of more than 30%. Phylogenetic analyses show the internal connection between the Middle East (Turkey, Iraq, Iran, Saudi Arabia), South Asia (India, Indonesia), and East Asia (China and Japan), while external viral communications might occasionally occur. In 18 strains with both sequential and mortality data, amino acid alignment showed several point substitutions that may be related to its virulence. We suggest more behavioral monitoring in Marek’s disease-endemic regions and further studies on strain virulence, together with its Meq protein structural changes.

## 1. Introduction

Marek’s disease (MD) is a threatening infectious disease for the poultry industry. MD was first reported in a chicken farm in Hungary in 1907 and rapidly spread around the world, with recorded mortalities ranging from 10 to 30% and occasionally above 60% in infected chickens. MD causes an economic loss of approximately USD 1 billion every year [1], despite efforts made in constructing vaccines and clinical monitoring [2,3]. The monitoring of MD in farms relies first on clinical pathology detection and is followed by genetic confirmation. Overall, the disease can be divided into two forms, neural and visceral types [4], with 10–25% and above 70% mortality, respectively. In neural MD, the main clinical symptoms include a complete or partial paralysis of the neck, wings, and limbs. Such paralyses are mainly induced by lesions of the vagus, brachial, and sciatic plexuses that show enlargement and yellowish color on the surface. In histopathological sections, vacuolar degeneration in nerve parenchyma and infiltration of mononuclear cells are usually observed, which indicates edema and inflammation. Sometimes in more severe infections, gray or cloudy eyes and small tumors in the ovary, heart, liver, lung, and other tissues can be found and, usually, iridocyclitis and lymphomas are further detected under microscopic observation. In visceral MD, the gross tumors can be observed in the gonads, liver, kidney, lung, heart, spleen, and proventriculus in larger sizes and higher numbers. These nodules are usually infiltrated with cancerous lymphoid cells [5,6,7]. In order for the disease to be better monitored, it is important to know which type is now dominant in outbreaks.

MD is caused by the infection with Marek’s disease virus (MDV) or known as *Gallid alphaherpesvirus 2* (GaHV-2) that belongs to the alphaherpesvirus, Mardivirus genus. MDV infection can be divided into three phases: the cytolytic phase, the latent phase, and the T-cell transformation phase. In the latter, MDV massively replicates in CD4+ T cells and induces oncogenic change to produce wide-spread tumorigenic T cells [8]. Importantly, the third stage is the one determining survival as the disease severity and consequent mortality are mainly decided by the efficiency in producing lymphoma cells (oncogenic transformation potential by MDV) and their transmitting potential [9]. The transformation potential has been studied at genetic level. The MDV genome consists of a unique long (UL) region flanked by inverted repeats, terminal repeat long (TRL), internal repeat long (IRL), and a unique short region (US) but flanked by inverted short repeats. The TRL locates on the initial left and right end of the genome, and it is found that a special 1020 bp long *meq* gene locates in the two regions and encodes a 339 aa long protein Meq [10]. The Meq contains a basic region-leucine zipper domain that is similar to Jun/Fos family activators. It was found that Meq can bind with c-Jun proteins or itself and form an heterodimer by this domain [11], and it will activate Jun, Fos, and ATF/CREB family proteins, resulting in upregulation of IL-2 and CD30 [12]. The two factors have been proven before to induce lymphomas in Hodgkin’s disease [13]. Moreover, cyclin-dependent kinase 2 is also induced after binding and can lead to dysregulation of cell cycle control by P53-RB pathway [14]. In addition, recent studies revealed that Meq can bind the C-terminal-binding protein (CtBp) [15] and the protein Myc, a master regulator of cell cycle entry and proliferative metabolism [16] protein. These two proteins are major regulators of viral telomerase RNA (vTR)-induced apoptosis, and the inhibition of their function by such binding with Meq will reduce tumor cells elimination. Accordingly, many studies have found that *meq* gene mutations result in a different virulences of MDV strains, and therefore *meq* sequence becomes an essential indicator of MDV virulence and strain classification [17]. 

Vaccination is a functional tool to protect chickens from developing MD syndromes and dying. In an early study, all three of the most well-known vaccines produced on the basis of strains SB-1, CV1988 (Rispens), and FC126 showed protective indexes of more than 90%, and these vaccines have reduced the outbreak times significantly around the world in the last 30 years [18]. However, some argue [19] that the persisting transmission of MDV in infected individuals may help in the evolution of MDV, possibly leading to the generation of more virulent strains with faster replication levels. In addition, further studies revealed that CVI1988 can no longer maintain its high efficiency towards certain new strains asking for an epidemiological investigation on the current status of MD in certain areas [20]. Asia is among the regions that face the most severe infections by MDV. In countries such as China, Japan, and India, the original harmful strains such as CVI1988 have a long history of infecting chickens, and identification of new virus strains is reported almost every year. Although vaccine injection, especially for CVI1988 and HVT, are also widely conducted in this region, additional studies have shown that the incidence rate of MD in Asia is increasing, and new outbreaks are usually linked to new virulent strains [21,22,23]. However, currently, the exact situation of MD in Asia for the past few years remains poorly understood. What are its disease pathology features? What is the virulence of recent MDV strains? How is sequence changes of *meq* in these strains? Moreover, how is the protection efficiency of commonly used vaccines? This study focused on available data in recent 10 years about these indexes and aimed at evaluating the need for renewing vaccines and strategies.

## 2. Materials and Methods

### 2.1. Literature Screening

The literature was reviewed on the basis of the four main databases, namely, Web of Science, PubMed, Scopus, and Google Scholar. The keywords used in this study were “Marek’s disease”, “Marek’s disease virus”, and “poultry” in Asia, and studies were screened to find records published between 2011 and 2021. We compiled studies including types of research, reviews, and case-reports that contain at least one piece of information about MD pathology, virus identification, virulence estimation, MD prevalence, and mortality in Asia. After that, every study compiled was checked for repeat cases and outbreaks. The final studies were then annotated for their study and outbreak year, region, infected farms, their MD incidence rate and mortality, vaccine situation, pathology types (visceral or neural types), isolation of viral strain, and identification method.

### 2.2. Phylogenetic Analyses

All recorded publications (29 out of 36 studies) with detailed *meq* sequence information or available viral strain *meq* information that were submitted to NCBI were exported to check for their accuracy and length according to the sequence information in tables and figures in order to avoid missing indel mutations before aligning using MEGA X. All sequences that showed apparent massive base pair absence or base pair inserting conflicting with the information in publications [24,25,26,27,28] were considered as error and were marked with “-“ or deleted, respectively, to reach the normal 1020 bp. It is likely that the error was generated through mis-uploading or data being missing. Then, aligned data were processed with both a bootstrap method (100 replication) for the test of phylogeny and a neighbor-joining method to calculate the evolutionary distances by R-4.1.2.

A total of 18 viral strains with both mortality rate and detailed *meq* sequence information were then selected for amino acid conversion and alignment on MEGA X. Twelve virulent strains with more than 30% morality rate and six less-virulent strains with less than 30% mortality rate were then checked for the amino acid substitutions in comparison to CVI988 strain before exporting the substitutions data in tables.

## 3. Results and Discussions

### 3.1. Overall MD Outbreaks in Asia between 2011 and 2021 and Associated Phylogeny Based on meq Gene

**Figure 1:**
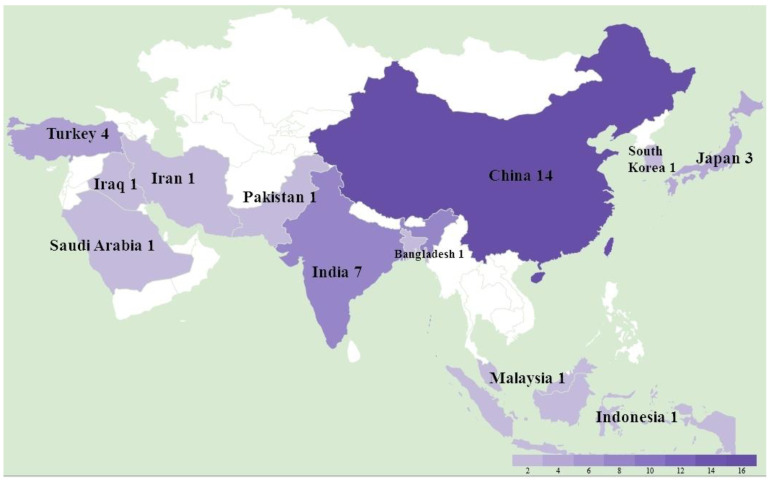
The study location and number of Marek’s disease outbreaks in Asia between 2011 and 2021.

**Figure 2:**
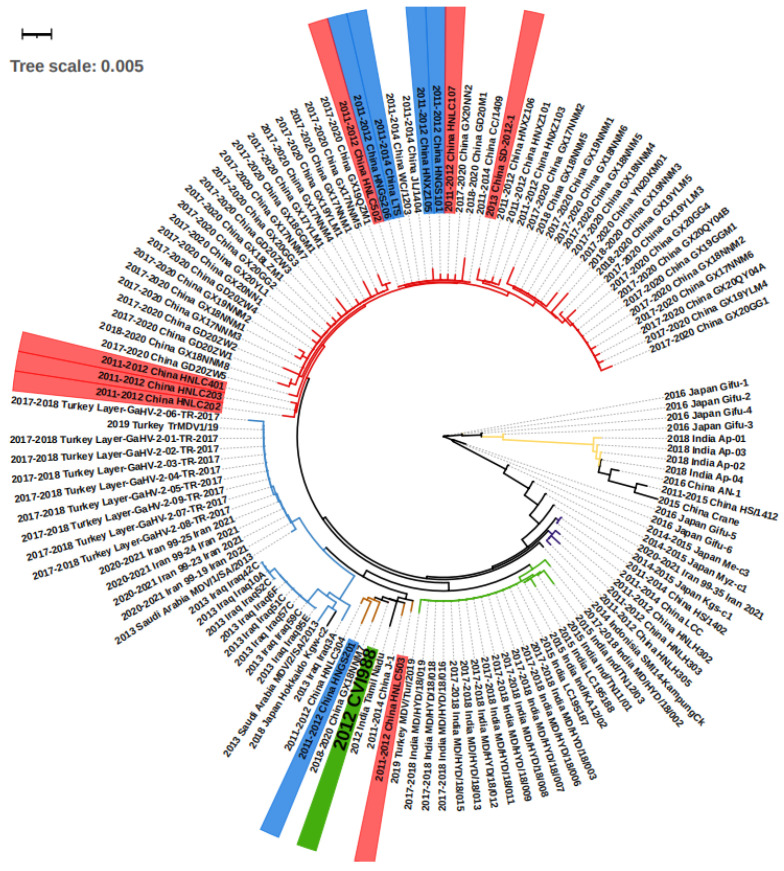
The phylogenetic tree of the isolated viruses according to *meq* sequences. On the strain names, green labels the vaccine strain CVI988, and blue and red label the strain that bypassed protection by the CVI988 vaccine, reaching a mortality of more than 30% and 50%, respectively. On the branches, red, brown, and purple label the China groups 1, 2, and 3, respectively. The blue, green, and yellow branches label the Middle East, India group 1, and India group 2, respectively. The *meq* consists of 1020 bp, and therefore 0.001 evolutionary distance can represent 1 different point mutation.

A total of 36 studies were recorded from 12 countries, namely, Bangladesh, China, India, Indonesia, Iran, Iraq, Japan, Malaysia, Pakistan, Saudi Arabia, South Korea, and Turkey (Figure 1). In detail, as shown in Table 1, a total of at least 289 farms were identified with MD outbreaks. Among 14 studies with pathological information, 4 studies reported a neural type while all studies reported some aspects of the visceral type. The lymphoma induced by MDV occured most frequently in the liver (11 studies), followed by spleen (9 studies), bursa of Fabricius (5 studies), kidney (4 studies), skin (4 studies), heart (4 studies), gizzard (4 studies), intestine (3 studies), and lung (2 studies), as shown in Table 2. In the four recorded neural types, lymphoma was detected in the sciatic nerves. MD incidence rates ranged from 0.1% to 40%, and MD mortality rates were distributed from 1% to 80% depending on the specific viral strains and vaccination status. Considering MDV strains and vaccination, 31 studies successfully isolated 132 different strains with *meq* sequential information, and 14 studies described the use of vaccines in farms including CVI988/Rispens (13 studies), HVT (4 studies), FC126 (3 studies), and two unknown vaccines. By either looking at the information from farms or following experimental research, we found that 12 strains from China were able to bypass the protection of the CVI988 vaccine, leading to a mortality of more than 30%. In seven cases, mortality reached more than 50% (Figure 2).

We conducted a phylogenetic analysis on the basis of the *meq* sequence of the 132 viruses, together with the CVI1988 strain, which is shown in Figure 2. Altogether, the majority of the viruses isolated from Middle Eastern countries including Turkey, Iraq, Iran, and Saudi Arabia showed very short distances with no more than 10 different base pairs, suggesting they may originate in the same ancestor. The strains found in Turkey, Iran, and Saudi Arabia were almost identical, with fewer than three base pair differences (Figure 2, Table S1). As an exception, the strain “2019 Turkey MDV/Tur/2019” was very close (fewer than 3 bp differences) to the virus “India Group 1” (green branch in Figure 2), which may suggest virus transmission between the two countries. The viruses in China can be mainly divided in three groups on the basis of sequence (Figure 2). First, we find the “China Group 1” (red branch in Figure 2) that dominated the most recent outbreaks. Second, the “China Group 2” (brown branch in Figure 2) that displayed a short distance (less than 3 different point mutations) with the vaccine strain CVI988. Finally, the third group included “China Group 3” (purple branch in Figure 2), which is closely related to “2014–2015 Japan Kgs-C1” and “2014 Indonesia SMI14-KampungCk”. Additionally, there are some exceptions: “2015 China Crane”, a strain isolated from a wild crane, which showed more similarities with “2011–2015 China HS/1412”, “2016 China An-1”, and the “India Group 2” (yellow branch, Figure 2) when compared with China Groups 1, 2, and 3, indicating that there may have been two branches of MDV evolution in China. The strains in India can be divided into two groups, one for the “India Group 1” (green branch in Figure 2), and the second group “India Group 2” (yellow branch in Figure 2). The first group was found to be closer to the “2015 China Crane branch”, and the second was closer to the “2014 Indonesia SMI14-KampungCk”. In Japan, except for the strains mentioned above, there were also “2016 Japan Gifu1-6” and “2014–2015 Japan Me-C3” that were found to only be poorly related to other strains (more than 10 different point mutations). Overall, according to the groups and evolutionary distances, we were able to divide the regions into three internal connected parts, the Middle East (Turkey, Iraq, Iran, Saudi Arabia), South Asia (India, Indonesia), and East Asia (China Group 3 and 2014–2015 Japan kgs-c1). The strains within the countries in the part showed less than 10 different point mutations. However, there was an additional “China Group 1” and various highly mutated strains that have mainly been found in Japan (right part of Figure 2).

### 3.2. MD in Specific Asian Countries

#### 3.2.1. China

In China, a total of at least 191 farms distributed in 17 provinces (8 in 14 China studies described the farm numbers) experienced a MD outbreak between 2011 and 2021 (Table 1). The deadliest cases came from the outbreak recorded in Shangdong (PRC) in 2013. It was described that the MD caused 38.3% mortality in a farm with 3000 chickens, even though they were vaccinated with FC126 at 1 day of age [30]. This study did not mention the overall MD incidence rate in the flock, but it is apparent that the rate would have been at least above 38.3%. The outbreak case reported in Jilin (PRC) in 2017 accounts for the most contagious case, with an MD incidence rate of 66.7%, despite protection with the vaccine CVI988 [40]. The best documented MD prevalence data, contributed by a continuous study between 2011 and 2015, described 165 MDV-positive farms in 12 provinces. In this study, the relatively low MD incidence and mortality rates of the HS/15 strain in unvaccinated conditions suggests that a group of infectious but not fatal MDVs are now spreading over China [45]. When considering pathological reports, tumor nodules are reported in all available four studies (Table 2). The most frequently found anatomic feature was the enlargement of the liver (four in four studies) associated with white nodules and infiltration of lymphocytes revealed by HE staining. This was associated with apparent diffused pleomorphic and neoplastic lymphocytes, leading to the destruction of the structure of the normal organs. In addition, similar tumor-associated changes were found in the heart (three in four studies), the spleen and gizzard (two in four studies), the kidney, the intestine, the muscles, and the bursa of Fabricius (one in four studies). It is interesting that in the case of the crane infection, the tumor invaded the trachea and may have caused respiratory issues.

Importantly, no study has described MD types with neural changes. One possible explanation is that all the detected samples came from dead chickens, which indicates that they would have suffered from the visceral type. In these studies, the behavioral changes usually shown in neural types were not continuously monitored. Thus, they may have missed the initial diagnosis time. However, we cannot exclude the possibility that now MDV strains have evolved to be more harmful and induce more visceral types than neural cases. When looking at MDV strains, a total of 71 strains with different *meq* sequences were isolated. Among them, most of the strains displayed similar *meq* mutations and strains isolated from the same province, and at similar times were closely related. According to the available data, the strain “SD-2012-1” should be the center of our attention, as it is close to recent 2017–2020 strains (Figure 2) and can induce MD with more than 50% of mortality under vaccine protection [30]. In addition, there are also six strains (HNLC202, 203, 401, 502, 503, 107) that have been reported to bypass the protection by the vaccine, leading to mortality levels of more than 50%, and five strains (LTS, HNGS101, 201, 206, HNXZ105) led to mortality levels ranging from 30% to 50% [31]. Compared with the CVI998 vaccine strain, the AN-1, HS/1412, and 2015 China Crane are highly mutated (Figure 2). More studies are needed for their epidemiological status and virulent studies. Overall, it is apparent that the most frequently used CVI988 and FC126 vaccines have started to be challenged over the last 10 years in China, especially in Henan and Shandong provinces. The renewal of vaccines could be an option for China, and the “SD-2012-1” strain may be helpful for the new vaccine development.

#### 3.2.2. India

According to the data available from seven studies, there are at least 25 confirmed farms distributed in eight states that suffered from Marek’s disease in the last decade (Table 1). The 2018 study in Meghalaya, India, recorded two fatal outbreaks, one with 5.5% mortality and the other with 34% mortality, despite the protection of a vaccine not described in detail [44]. In an investigation of an area in Andhra Pradesh, India, where MD was suspected, a total of 27 chicken blood samples from live chickens and 84 tissue samples from dead chickens were analyzed, and all tested positive for MDV detection. However, the authors did not publish the exact incidence rate of MD [26]. When considering the pathological characteristics, we found the visceral type in almost every study that considered dead farm chickens. Liver lymphomas were found in three out of the four studies, followed by lung tumors in two studies. Other organs including kidney, spleen, heart, skin, and gizzard were also found with tumors each in one of the studies. It should be noted that sciatic nerve was also recorded with lymphoma in a study [44]. The detailed pathological changes included the enlargement of organs associated with abnormal structure and white nodules, as well as the identification by HE staining of lymphoblastic tumors. A study using field isolated viruses to infect experimental unvaccinated chickens showed an early paralysis of limbs, unilateral thickening of the sciatic nerve well before 12 weeks of age, followed by more serious visceral lesions and tumors [36]. Thus, it suggests that neural symptoms occur early in the outbreak, and monitoring the initial neural types in a farm might help in preventing a more serious outbreak. In these studies, a total of 23 strains with different *meq* sequences were isolated. The viruses within “India group 1” (green branch in Figure 2) showed no more than five point *meq* genetic mutations and were less closely related to “India group 2” (yellow branch in Figure 2) virus strains with more than 15 point mutations. The 2015 India “LC195187” and 2015 India “LC198188” are the two most deadly viruses according the data described. They induced a mortality of 34% MD in farms, although chickens were vaccinated with an unknown vaccine. The majority of strains (20 in 23) had no virulence data. The vaccine used in India was mainly HVT, which generated significant protection (decreasing the mortality rate from 40% to 10% and MD incidence rate from 100% to 57.5%) for 2015 India Ind/TN12/03 [36]. Therefore, it seems this vaccine is efficient. However, we still need more evidence for other groups, especially AP strain studies.

#### 3.2.3. Japan

Four studies described the outbreak of Marek’s disease in seven farms, but none of them described prevalence or mortality data (Table 1). The chickens for the study performed in Gifu, Japan, in 2016 were vaccinated with CVI988, but this still did not prevent the outbreak [24]. Chickens infected with MD showed apparent visceral lesions with lymphomas in the liver, spleen, intestine, kidney, and gizzard. Neural changes were also found with oedema, loss of striation, and discoloration in their peripheral and sciatic nerves [24]. A total of 10 strains were isolated, and the analysis of *meq* mutations showed diversity according to their evolutionary distances. The 2016 Japan Gifu-1, -2, -3, and -4 were the most distantly related to the 2018 Japan Hokkaido kgw-c2. The remaining five strains are more similar to each other but also show a more distant relationship with the 2016 Japan Gifu1-4 and 2018 Japan Hokkaido kgw-c2 (Figure 2). Although the outbreak in farms is now frequently reported in Japan, the diversity may suggest that MDV has a long evolution history there.

#### 3.2.4. Turkey

Turkey is among the countries that suffers from Marek’s disease outbreak. A total of 62 farms have been reported to have MD in four studies (Table 1). Two studies [25,41] isolated MDV in 206 from different chickens in 752 diseased spleen and blood samples. A study conducted in a farm with 10,000 chickens showed a mortality rate between 1% and 2%, despite the protection by vaccines CVI988, HVT, and FC-126 [53]. According to two studies [41,53] that investigated the pathological changes, the visceral lesions were described with liver and spleen swelling and lymphomas. In addition, the heart, kidney, and gizzard were also found in either one of the two studies, and one study recorded the neural type with sciatic nerve tumor [41]. Cases included 11 viral strains in the three studies, but most of them showed the same *meq* sequence, including the one with available mortality data, except for 2017–2018 Turkey Layer-GaHV-2-06-TR-2017 showing three point mutations (Figure 2, Table S1). Thus, the Marek’s disease situation may be protected well with the vaccines in Turkey, but the risk for potential transmission and evolution still exists.

#### 3.2.5. Other Regions

In addition to the countries described above, there are also single reports for Bangladesh, Indonesia, Iraq, Iran, Malaysia, Pakistan, Saudi Arabia, and South Korea in the last decade. In the Iraqi study, researchers bought chickens from farms and markets and found an overall prevalence of 49.5% MDV, although they did not mention whether these chickens were selected on the basis of behavioral performance [27]. In Bangladesh, among the country-wide collection of dead chickens, 4710 chickens were analyzed, and 6.69% of them died with MD [33]. It is surprising that MDV was successfully isolated from 2 out of 16 *Dermanyssus* *gallinae* mites, suggesting that parasites may be able to transmit this virus [47]. Studies in Saudi Arabia showed that the vaccines CVI998, HVT, and FC-126 failed to prevent the transmission of MD in farms, although with a low mortality around 10% [39]. There are three studies recording the pathological types, and all of them documented a visceral type, except for the study in Bangladesh, which also showed neural changes. As shown in Table 2., two studies describe tumors in the bursa of Fabricius and spleen, while each of the three studies showed lymphomas in the liver, skin, and gizzard. Specifically, 110 birds had complete paralysis of wings and legs and diarrhea, and 52 had partial paralysis of wings in the farms found in Bangladesh [33]. For the viruses, nine strains in Iraq, five in Iran, two in Saudi Arabia, and one in Indonesia were isolated. The strains within the same studies are similar in meq mutations in Iraq and Iran, except for 2013 Iraq3A, which has more mutations when compared with others in Iraq (Figure 2, Table S1).

### 3.3. Amino Acid Changes of Virulent Strains

As labeled in Figure 1, there were 12 virulent strains with sequential information that can bypass the CVI988 vaccine. The mortal rates of these viruses were all above 30%. In order to discover virulence-associated amino acid changes, we aligned amino acid sequences of the 12 strains with CVI988 sequence and found candidate changes, as shown in Table 3. Overall, we found a total of 17 amino acid site substitutions. The S to A substitution at site 71, D to Y at site 80, V to A at site 115, P to R at site 176, and P to A site at site 217 were the most common ones, and they coexisted in 11 strains. Occasionally, substitutions were shown from site 27 to 332. The Meq can be divided into an N-terminal basic region (BR) (from site 30 to 85) and a leucine zipper (ZIP) domain (from site 85 to 121), as well as a C-terminal proline-rich transcriptional regulatory domain structurally (from site 121 to 339) [11]. These domains account for nucleolar localization, oncogenic protein binding, and transcription regulations [56], respectively. According to a previous study regarding the the A substitution at 71 sites, proline rich sites change at sites 176 and 217 are positively related to virulent increase [57]. This phenomenon was also found in this study. The D to Y at site 80 and V to A substitutions at site 115 were widely found in China and were suggested to be used as a virulent strain label in a 2011 study [58]; our review also supports this idea. However, the current knowledge for how the amino acid substitution changed its virulence is still lacking. As researches revealed that the Meq can bind directly to oncogenic-associated proteins such as Jun and P53 [59], we suggest the conducting of more studies on how amino acid substitutions affect these bindings and final virulence, beginning with the substitutions reviewed in this study.

Additionally, we aligned the six less-virulent strains that cannot bypass the CVI988 vaccine with less than 30% mortality rate. The results are shown in Table 4. A total of 16 types of amino acid substitutions were found, and the same types of 71, 80, 115, 139, 176, and 217 site changes as the ones in the 12 virulent strains occurred in five China strains. The “2019 Turkey MDV/Tur/2019” showed different amino acids with the virulent strains at the sites 115(L), 139(A), 176(p), and 217(p). When comparing amino acid substitution at the site 139 between all 18 strains, we found that all six less virulent and five virulent (30% to 50% mortality) showed T to A substitution, whereas five in seven very virulent strains (more than 50% mortality) showed no substitution. Moreover, four in six less virulent strains showed A to substitution at site 88 and Q to R substitution at site 93. It is likely that such substitutions on sites 88, 93, and 139 are associated with less virulence. More studies on the impact on the Meq structure and binding functions will be helpful.

## 4. Conclusions

In conclusion, in the last decade, Marek’s disease was being transmitted in 12 Asian countries, and we think that more undocumented outbreaks may occur in associated regions. The visceral type dominated the pathological detection outcomes, but the initial neural disease may also potentially occur. We suggest more behavioral monitors in epidemic countries. According to *meq* genetic sequences, the new strains are similar within the Middle East, South Asia, and East Asia regions, while some external communications exist. In particular, China may now consider renewing the CVI988 vaccination as the outbreak time and virulent strain reports are frequent. The amino acid substitutions at sites 71 (S to A), 80 (D to Y), 115 (V to A), 176 (P to R), and 217 (P to A) are common in virulent strains and have been proven to be associated with increased virulence. The amino acid substitutions at 88 (A to T), 93 (Q to R), and 139 (T to A) may be related to virulence decrease. However, all these substitutions are needed to be studied in structural and infection studies for final results.

## Figures and Tables

**Table 1:** Marek’s disease outbreak and its pathogenicity features in Asia between 2011 and 2021.

Year of Study	Outbreak Year	Region	Number of Chicken Farms	MD Incidence Rate in Farm	Mortality Rate in Farm	Vaccine	Pathology Types	Number of Strains	Method	References
2012	*	India,	*	*	*	*	Visceral	1	PCR, pathology	[29]
2013	2013	China	1	*	38.30%	FC126	Visceral	1	PCR, pathology	[30]
2013	2011–2012	China	18	*	*	*	*	17	PCR	[31]
2013	*	India	12	*	*	unknown vaccine	Visceral	*	Pathology	[32]
2013	2011	Bangladesh	*	*	*	*	Visceral	*	Pathology	[33]
2013	2013	Iraq	*	*	36.8% and 65%	*	*	9	PCR	[27]
2015	2011	China	*	*	*	*	*	*	PCR	[34]
2015	2014	China	1	5%	80%	CVI988/Rispens	Visceral	*	PCR, pathology	[35]
2015	2012	India	5	*	*	*	Visceral	*	PCR, pathology	[36]
2016	2013	China	1	*	22.30%	CVI988/Rispens	*	*	PCR	[37]
2016	2014	Indonesia		38.8%, 35%, and 20.8%	*	*	*	1	PCR	[38]
2016	2016	Saudi Arabia	*	*	*	CVI988, HVT and FC126		2	PCR	[39]
2017	2011–2014	China	*	20–40%	*	CVI988/Rispens	*	6	PCR	[21]
2017	2015	China	1	36%	*	CVI988/Rispens	*	1	PCR	[40]
2017	2014	Japan	*	*	*	*	*	3	PCR, pathology	[28]
2017	*	Turkey	11	*	*	*	Neural, visceral	*	PCR, pathology	[41]
2018	2015	China	*	*	*	*	Visceral	1	PCR, pathology	[42]
2018	2016	China	1	0.10%	*	CVI988/Rispens		1	PCR	[43]
2018	*	India	*	*	*	*	*	*	PCR	[26]
2018	2015	India	2	*	5.5% and 34%	unknown vaccine	Neural, visceral	5	PCR, pathology	[44]
2018	2016	Japan	6	*	*	CVI988 and HVT	Neural, visceral	6	PCR, pathology	[24]
2019	2011–2015	China	165	*	*	*	*	1	PCR	[45]
2019	2017–2018	Turkey	49	*	*	CVI988 and HVT	*	9	PCR	[25]
2019	2018	Malaysia	4	*	*	*	Visceral	*	PCR	[46]
2020	2018–2019	South Korea	*	*	*	*	*	*	PCR	[47]
2021	2017–2020	China	*	*	*	*	*	37	PCR	[48]
2021	2018	China	3	20%	10%	CVI988/Rispens	Visceral	1	PCR, pathology	[49]
2021	2018–2020	China	*	5–20%	2–10%	CVI988/Rispens	*	5	PCR	[50]
2021	2017–2018	India	5	*	*	*	*	13	PCR	[23]
2021	2018	India	1	*	*	*	*	4	PCR	[22]
2021	2018	Japan	1	*	*	*	*	1	PCR, pathology	[51]
2021	2019	Turkey	1	*	*	*	*	1	PCR	[52]
2021	2019	Turkey	1	*	1–2%	CVI988, HVT and FC126	Visceral	1	PCR, pathology	[53]
2021	2019	Pakistan	*	*	*	*	Neural, visceral	*	Pathology	[54]
2021	2020–2021	Iran	*	*	*	*	*	5	PCR	[55]

Note: the ‘*’ represents no available data in the study.

**Table 2:** Organs in which tumors were found in visceral MD.

References	Region	Species	Liver	Spleen	Bursa of Fabricius	Kidney	Skin	Heart	Gizzard	Intestine	Lung
[35]	China	layer	y	y	y	*	toe	*	*	*	*
[42]	China	Crane	y	*	*	*	leg	y	y	*	*
[30]	China	broiler	y	y	*	y	*	y	y	*	*
[49]	China	broiler	y	*	*	*	*	y	*	y	*
[29]	India	layer	y	*	y	*	*	*	*	*	*
[32]	India	layer	*	*	*	*	leg	*	*	*	y
[36]	India	layer	y	y	y	*	*	*	*	y	*
[44]	India	layer	y	y	*	y	*	*	*	*	y
[41]	Turkey	layer	y	y	*	y	*	*	y	*	*
[53]	Turkey	broiler	y	y	*	*	*	y	*	*	*
[24]	Japan	broiler	y	y	*	y	*	*	y	y	*
[33]	Bangladesh	broiler	*	*	y	*	y	*	*	*	*
[46]	Malaysia	broiler	y	y	y	*	*	*	*	*	*
[54]	Pakistan	broiler	*	y	*	*	*	*	*	*	*

Note: * represents no available data in the study; “y” represents the tumor was identified in the organs.

**Table 3:** Amino acid substitutions of 12 virulent MDV.

Viral Strain	Amino Acid Site																
	27	59	71	80	88	115	133	139	176	217	237	254	277	293	318	320	332
2012 CVI988	S	K	S	D	A	V	T	T	P	P	C	I	L	P	I	I	W
2011–2012 China HNXZ105	.	.	A	Y	.	A	P	A	R	A	.	.	.	.	.	.	.
2011–2012 China HNXZ101	.	.	A	Y	T	A	.	A	R	A	.	.	.	L	.	.	.
2011–2014 China LTS	.	.	A	Y	.	A	.	A	R	A	.	.	.	.	.	.	.
2011–2012 China HNGS206	.	.	A	Y	.	A	.	A	R	A	.	.	.	.	.	.	.
2011–2012 China HNGS201	.	E	.	.	.	.	.	.	.	.	S	V	.	.	.	M	.
2011–2012 China HNLC503	.	.	.	.	.	.	.	.	.	A	S	.	.	.	.	.	.
2011–2012 China HNLC401	P	.	A	Y	.	A	.	.	R	A	.	.	.	.	.	.	.
2011–2012 China HNLC502	.	.	A	Y	.	A	.	A	R	A	.	.	.	.	.	.	.
2011–2012 China HNLC203	.	.	A	Y	.	A	.	.	R	A	.	.	.	.	.	.	.
2011–2012 China HNLC202	.	.	A	Y	.	A	I	.	R	A	.	.	.	.	.	.	G
2011–2012 China HNLC107	.	.	A	Y	.	A	.	A	R	A	.	.	F	.	.	.	.
2013 China SD-2012-1	.	.	A	Y	T	A	.	.	R	A	.	.	.	.	V	.	.

Note: ‘.’ means the amino acid type in this site was the same as the type in the same site of CVI988 amino acid sequence. The very virulent strains with more than 50% mortality and the most commonly found substitutions are labeled in red.

**Table 4:** Amino acid substitutions of 6 less virulent MDV.

Viral Strain	Amino Acid Site															
	2	32	39	69	71	80	88	93	112	115	139	176	217	234	249	340
2012 CVI988	S	K	I	D	S	D	A	Q	S	V	T	P	P	P	P	-
2018 China GX18NNM5	A	.	.	.	A	Y	T	R	.	A	A	R	A	.	.	.
2018–2020 China GX18NNM8	A	.	.	.	A	Y	.	.	.	A	A	R	A	.	T	R
2018–2020 China GX19YLM5	.	.	.	.	A	Y	T	R	.	A	A	R	A	.	.	.
2018–2020 China GX19NNM3	.	R	.	G	A	Y	T	R	P	A	A	R	A	.	.	.
2018–2020 China GD20M1	.	.	V	.	A	Y	.	.	.	A	A	R	A	L	.	.
2019 Turkey MDV/Tur/2019	-	-	.	.	A	Y	T	R	.	L	A	.	.	.	.	.

Note: “.“ means the amino acid type in this site is the same as the type in the same site of CVI988 amino acid sequence. The most commonly found substitutions are labeled in red.

## Data Availability

All genetic data set in this study can be found at http://www.NCBI.com (accessed on 1 December 2021) or in the references. The references cited in this study are from Web of Science, PubMed, Scopus, and Google Scholar.

## Supplementary Materials

The following supporting information can be downloaded at https://www.mdpi.com/article/10.3390/ani12050540/s1, Table S1: *meq* sequential information of MDV strains used in this study.
